# A multiethnic genome-wide analysis of 19,420 individuals identifies novel loci associated with axial length and shared genetic influences with refractive error and myopia

**DOI:** 10.3389/fgene.2023.1113058

**Published:** 2023-06-07

**Authors:** Chen Jiang, Ronald B. Melles, Jie Yin, Qiao Fan, Xiaobo Guo, Ching-Yu Cheng, Mingguang He, David A. Mackey, Jeremy A. Guggenheim, Caroline Klaver, Consortium for Refractive Error and Myopia (CREAM), K. Saidas Nair, Eric Jorgenson, Hélène Choquet

**Affiliations:** ^1^ Division of Research, Kaiser Permanente Northern California (KPNC), Oakland, CA, United States; ^2^ KPNC, Department of Ophthalmology, Redwood City, CA, United States; ^3^ Centre for Quantitative Medicine, Duke-NUS Medical School, Singapore, Singapore; ^4^ Ophthalmology and Visual Sciences Academic Clinical Program (Eye ACP), Duke-NUS Medical School, Singapore, Singapore; ^5^ Department of Statistical Science, School of Mathematics, Sun Yat-Sen University, Guangzhou, China; ^6^ Southern China Center for Statistical Science, Sun Yat-Sen University, Guangzhou, China; ^7^ Ocular Epidemiology Research Group, Singapore Eye Research Institute, Singapore, Singapore; ^8^ State Key Laboratory of Ophthalmology, Zhongshan Ophthalmic Center, Sun Yat-Sen University, Guangzhou, China; ^9^ Centre for Eye Research Australia; Ophthalmology, Department of Surgery, University of Melbourne, Melbourne, WA, Australia; ^10^ Lions Eye Institute, Centre for Ophthalmology and Visual Science, University of Western Australia, Perth, WA, Australia; ^11^ School of Optometry and Vision Sciences, Cardiff University, Cardiff, United Kingdom; ^12^ Department Ophthalmology, Department Epidemiology, Erasmus Medical Center, Rotterdam, Netherlands; ^13^ Department of Ophthalmology and Department of Anatomy, School of Medicine, University of California, San Francisco, CA, United States; ^14^ Regeneron Genetics Center, Tarrytown, NY, United States

**Keywords:** GWAS, genetics, single nucleotide polymorphisms (SNPs), axial length, eye biometry, refractive errors, myopia

## Abstract

**Introduction:** Long axial length (AL) is a risk factor for myopia. Although family studies indicate that AL has an important genetic component with heritability estimates up to 0.94, there have been few reports of AL-associated loci.

**Methods:** Here, we conducted a multiethnic genome-wide association study (GWAS) of AL in 19,420 adults of European, Latino, Asian, and African ancestry from the Genetic Epidemiology Research on Adult Health and Aging (GERA) cohort, with replication in a subset of the Consortium for Refractive Error and Myopia (CREAM) cohorts of European or Asian ancestry. We further examined the effect of the identified loci on the mean spherical equivalent (MSE) within the GERA cohort. We also performed genome-wide genetic correlation analyses to quantify the genetic overlap between AL and MSE or myopia risk in the GERA European ancestry sample.

**Results:** Our multiethnic GWA analysis of AL identified a total of 16 genomic loci, of which 5 are novel. We found that all AL-associated loci were significantly associated with MSE after Bonferroni correction. We also found that AL was genetically correlated with MSE (r_g_ = −0.83; SE, 0.04; *p* = 1.95 × 10^−89^) and myopia (r_g_ = 0.80; SE, 0.05; *p* = 2.84 × 10^−55^). Finally, we estimated the array heritability for AL in the GERA European ancestry sample using LD score regression, and found an overall heritability estimate of 0.37 (s.e. = 0.04).

**Discussion:** In this large and multiethnic study, we identified novel loci, associated with AL at a genome-wide significance level, increasing substantially our understanding of the etiology of AL variation. Our results also demonstrate an association between AL-associated loci and MSE and a shared genetic basis between AL and myopia risk.

## Introduction

Ocular axial length (AL) is an eye dimension that refers to the distance (in millimeters) from the apex of the anterior corneal (front of the eye) to the retina. AL elongation is a feature of myopia, which is the most common form of refractive error, and several other eye diseases such as retinal tear, retinal detachment, inherited retinal diseases, glaucoma, and myopic macular degeneration (Adams, 1987; Grosvenor, 1988; Arias et al., 2015; Tideman et al., 2016; Tideman et al., 2018; Streho et al., 2019; Rezapour et al., 2021; Fonteh et al., 2022; Foo et al., 2022; Omodaka et al., 2022; Williams et al., 2022; Xu et al., 2022). Moreover, the measurement of AL is generally more precise and reproducible than assessments of refractive/myopia status (Mu et al., 2022). For these reasons, investigation of factors that influence the variation of AL endophenotype is relevant and could provide key insights into the etiology of eye diseases.

AL is predominantly genetically determined, with heritability estimates up to 0.94 (Biino et al., 2005; Dirani et al., 2006; Chen et al., 2007; Paget et al., 2008; Klein et al., 2009; Guggenheim et al., 2013). Although genome-wide association studies (GWAS) have considerably facilitated a wide range of discoveries in the human genetics (Visscher et al., 2017), few GWAS of AL have been reported to date (Fan et al., 2012; Cheng et al., 2013; Miyake et al., 2015; Fuse et al., 2022). Those GWAS were conducted in either European ancestry or in Asian ancestry populations. A recent study (Fan et al., 2020) investigating the genetic basis of corneal curvature identified eight associations with AL in a subset of the Consortium for Refractive Error and Myopia (CREAM) cohorts of European or Asian ancestry. To our knowledge, no studies have yet reported a GWAS of AL in a large and ethnically diverse cohort. It is also unclear what is the shared genetic background between AL, spherical equivalent (that quantifies refractive error), and myopia. Therefore, there is a clear need for research to illuminate the genetic underpinnings of AL.

In this study, we conducted a large multiethnic GWA meta-analysis of AL, including 19,420 individuals from the Genetic Epidemiology Research on Adult Health and Aging (GERA) cohort, with validation of the top associated single nucleotide polymorphisms (SNPs) (*p* < 5.0 × 10^−8^) at each locus in 10,851 participants from the CREAM cohorts. We subsequently fine-mapped these AL associations and prioritized causal genes and biological pathways. We then undertook a multiethnic GWA analysis of mean spherical equivalent (MSE) in 72,388 GERA participants and examined associations of lead AL-associated SNPs with MSE. Finally, we investigated the shared genetic effects between AL loci and MSE or myopia.

## Methods

### GERA cohort and AL measurement

The GERA cohort comprises 110,266 adult men and women who are consented participants in the Research Program on Genes, Environment, and Health, established for members of the Kaiser Permanente Medical Care Plan, Northern California Region (KPNC) (Banda et al., 2015; Kvale et al., 2015). The current study population included 19,420 GERA participants, 18 years and older, who were of non-Hispanic white, Hispanic/Latino, Asian, or African American race/ethnicity, and who had at least one recorded AL measurement on both eyes during the same visit (Table 1). In KPNC, biometry measurements, including AL, are collected using the Haag-Streit Lenstar 900 device prior to cataract surgery. The mean (in millimeters) AL of an individuals’ two eyes was used for the analyses. All study procedures were approved by the Institutional Review Board of the Kaiser Foundation Research Institute.

**TABLE 1:** Characteristics of GERA subjects included in the current study by sex, and ethnic group.

		N	AL (mm) Mean ± SD	MSE (diopters), Mean ± SD	Age at 1st AL measurement (years), Mean ± SD
**N**		19,420	24.07 ± 1.32	−0.38 ± 2.66	75.1 ± 8.7
**Myopia**					
Cases		5,448	25.02 ± 1.28	−3.13 ± 2.30	73.0 ± 8.7
Controls		9,846	23.51 ± 0.93	1.14 ± 1.27	76.3 ± 8.4
**Sex**	Female	11,501	23.86 ± 1.30	−0.32 ± 2.70	74.5 ± 8.7
	Male	7,919	24.38 ± 1.28	−0.48 ± 2.59	75.9 ± 8.8
**Ethnicity**	NHW	16,523	24.01 ± 1.26	−0.30 ± 2.59	75.4 ± 8.6
	H/L	1,209	24.01 ± 1.35	0.00 ± 2.52	73.8 ± 9.3
	EAS	1,209	24.88 ± 1.75	−1.94 ± 3.31	72.5 ± 9.4
	AA	479	24.12 ± 1.26	−0.28 ± 2.50	73.4 ± 9.0

Abbreviations: N, number of participants; SD, standard deviation; MSE, mean spherical equivalent; NHW, non-Hispanic white people; H/L, Hispanic/Latinos; EAS, east asians; AA, African-Americans.

### Genotyping and imputation

GERA DNA samples were genotyped at the Genomics Core Facility of the University of California, San Francisco (UCSF) on four ancestry-specific Affymetrix Axiom arrays (Affymetrix, Santa Clara, CA, USA) optimized for individuals of European, Latino, East Asian, and African American ancestry (Hoffmann et al., 2011a; Hoffmann et al., 2011b). Genotype QC (quality control) procedures were performed on an array-wise basis (Kvale et al., 2015), as follows: SNPs with initial genotyping call rate ≥97%, allele frequency difference (≤0.15) between males and females for autosomal markers, and genotype concordance rate (>0.75) across duplicate samples were included.

Imputation was also conducted on an array-wise basis. For imputation, we additionally removed variants with call rates <90%. Genotypes were pre-phased with Eagle (Loh et al., 2016) v2.3.2, and then imputed with Minimac3 (Das et al., 2016) v2.0.1, using two reference panels: variants were preferred if present in the EGA release of the Haplotype Reference Consortium (HRC; n = 27,165; no indels) reference panel (McCarthy et al., 2016), and from the 1000 Genomes Project Phase III release if not (n = 2,504; including indels) (Birney and Soranzo, 2015). As a QC metric, we used the info *r* ^2^ from IMPUTE2, which is an estimate of the correlation of the imputed genotype to the true genotype. We considered variants with a good imputation score if *r* ^2^ ≥ 0.7.

### GWAS analyses of AL in GERA

Each of the four GERA ethnic groups (non-Hispanic white, Hispanic/Latino, East Asian, and African-American) were first analyzed individually. We performed a linear regression of AL and each SNP using PLINK (Chang et al., 2015) v1.90 (www.cog-genomics.org/plink/1.9/) with the following covariates: age, sex, and ancestry principal components (PCs). The top ten ancestry PCs and the percentage of Ashkenazi (ASHK) ancestry were included as covariates for the non-Hispanic white ethnic group, while the top six ancestry PCs were included for the three other ethnic groups (Banda et al., 2015). The genomic inflation factor λ was calculated for each GWAS analysis to assess inflation due to population stratification (Yang et al., 2011). Data from each genetic variant were modeled using additive dosages to account for the uncertainty of imputation (Huang et al., 2009). The GWAS analyses were also conducted using a recent approach accounting for relatedness that-fits a whole-genome regression model, implemented in REGENIEv2.0.2 (Mbatchou et al., 2021). This new machine-learning method requires only local segments of the genotype matrix to be loaded in memory, making it faster and more efficient in memory use compared to other methods.

We then performed a meta-analysis of AL in GERA by combining the results of the four ethnic groups using the package “meta” of R (https://www.R-project.org) (Balduzzi et al., 2019). Fixed effects and random effects summary estimates were calculated for an additive model. Heterogeneity index, *I* ^2^ (0%–100%) and *p*-value for Cochrane’s Q statistic among ethnic groups were assessed. For each locus, the lead SNP was defined as the most significant variant within a 2 Mb window, and novel loci were defined as those that were located over 1 Mb apart from any previously reported locus.

### Replication of novel AL-associated loci in CREAM

To test the 5 novel AL-associated SNPs identified in the current study for replication, we evaluated associations in a subset of CREAM participants with AL measurement (Fan et al., 2020). GWAS summary statistics for AL from the study of Fan et al. (Fan et al., 2020), consisting of 10,851 individuals of European and Asian descent from 9 studies, were used for those replication analyses. Among the 5 novel AL-associated SNPs identified in the current study, 2 were available in the CREAM GWAS summary statistics; thus, the Bonferroni threshold was set up at a *p* ≤ 0.025 (to account for a total of 2 SNPs tested) for associations that replicated. However, the 5 novel loci were visually inspected by generating regional plots using the European descent CREAM dataset. To generate those regional plots, we used LocusZoom web-based plotting tool (Pruim et al., 2010; Boughton et al., 2021).

### COJO (conditional) analysis

To potentially identify independent signals within the 15 identified genomic regions, we performed a multi-SNP-based conditional and joint association analysis (COJO) (Yang et al., 2012), which is implemented in the Genome-wide Complex Trait Analysis (GCTA) integrative tool (Yang et al., 2013). This COJO analysis was conducted on the GERA non-Hispanic white ethnic group GWAS analysis results. To calculate linkage disequilibrium (LD) patterns we used 10,000 random samples from GERA non-Hispanic white ethnic group as a reference panel. For this COJO analysis, a *p*-value less than 5 × 10^−8^ was considered significant.

### AL array heritability

Array heritability estimate was obtained for AL in GERA non-Hispanic white people (the largest ethnic group of GERA) using LD score regression (Zheng et al., 2017).

### Variants prioritization

To prioritize variants within the 15 AL-associated loci identified in the GERA non-Hispanic white ethnic group GWAS analysis, we used a Bayesian approach (CAVIARBF) (Chen et al., 2015). For each of the 15 associated signals, we computed each variant’s capacity to explain the identified signal within a 2 Mb window (±1.0 Mb with respect to the lead SNP). Then, the smallest set of SNPs that included the causal variant with 95% probability (95% credible set) was derived. For this CAVIARBF analysis, we used 10,000 random samples from the GERA non-Hispanic white ethnic group as a reference panel to calculate LD patterns.

### VEGAS2 gene-based and pathway analyses

To prioritize genes and biological pathways, we conducted gene-based and pathways association analysis using the Versatile Gene-based Association Study - 2 version 2 (VEGAS2v02) web platform (Mishra and Macgregor, 2015). We first performed a gene-based association analysis on the GERA non-Hispanic white ethnic group GWAS analysis results using the default ‘-top 100’ test that uses all (100%) variants assigned to a gene to compute gene-based *p*-value. We used 1000 Genomes phase 3 European population as the reference panel. As 20,950 genes were tested, the *p*-value adjusted for Bonferroni correction was set as *p* < 2.28 × 10^−6^ (0.05/20,950).

Second, we performed a pathway association analysis based on VEGAS2 gene-based *p*-values. We tested enrichment of the genes defined by VEGAS2 in 9,734 pathways or gene-sets derived from the Biosystem’s database (https://vegas2.qimrberghofer.edu.au/biosystems20160324.vegas2pathSYM). We adopted the resampling approach to perform this pathway analysis using VEGAS2 derived gene-based *p*-values considering the default ‘−10 kbloc’ parameter as previously described (Iglesias et al., 2018). As 9,734 pathways or gene-sets were tested, the *p*-value adjusted for Bonferroni correction was set as *p* < 5.14 × 10^−6^ (0.05/9,734).

### Association analysis of lead AL-SNPs with MSE

We evaluated the associations of the 17 lead AL-associated SNPs (sixteen from the multiethnic meta-analysis and an additional one from the non-Hispanic white ethnic group GWA analysis) with MSE by linear regression under an additive model, and adjusting for age, sex, and ancestry PCs. As previously described (Hysi et al., 2020; Choquet et al., 2022), GERA individuals had at least one spherical equivalent value measured during routine eye examinations. Most subjects had multiple measures for both eyes. Spherical equivalent was calculated as the sphere + (cylinder/2). The spherical equivalent was selected from the first documented refraction assessment, and the mean of both eyes was used. After excluding participants with histories of cataract surgery (in either eye), refractive surgery, keratitis or corneal diseases, our GERA sample for this analysis included 72,388 individuals with spherical equivalent measurement from four ethnic groups (Supplementary Table S1). As a note, among those with spherical equivalent measurement, 15,503 (79.8%) were included in the GERA AL sample.

### Genetic correlation between AL and MSE or myopia

LD score regressions (Bulik-Sullivan et al., 2015) were conducted, using the LDSC v1.0.1 command line tool (https://github.com/bulik/ldsc), to estimate the genome-wide genetic correlations (*r*_g_) between AL and MSE and between AL and myopia. We used as input data GWAS summary statistics from our previous GWASs of MSE and myopia conducted in the GERA cohort (Choquet et al., 2022) (including 59,094 individuals with RE measurement, and 19,540 myopia cases and 36,487 controls of non-Hispanic white ethnic group). As previously described (Choquet et al., 2022), in GERA, myopia cases were defined as having a MSE ≤ -0.75 diopters (D) and controls as having a MSE > -0.75 D, corresponding to the definition of ‘any myopia’, as previously used (Hysi et al., 2020). This more myopic threshold (compared to the well-accepted threshold for myopia of MSE ≤ −0.50 D), increases the probability that only ‘‘true’’ myopes are included (Flitcroft et al., 2019).

## Results

### GERA cohort and AL

The GERA cohort is an unselected cohort of adult members of the KPNC integrated healthcare delivery system, with ongoing longitudinal records from vision examinations. For this study, our GERA sample consisted of 19,420 individuals from 4 ethnic groups (85.1% non-Hispanic white ethnic group, 6.2% Hispanic/Latino, 6.2% East Asian, and 2.5% African American) with a measured AL (Table 1). In our GERA sample, East Asian individuals had higher ALs on average than other groups, consistent with previous studies (Ip et al., 2007; Knight et al., 2012).

### Multiethnic genome-wide association study (GWAS) of AL in 19,420 GERA participants

**FIGURE 1:**
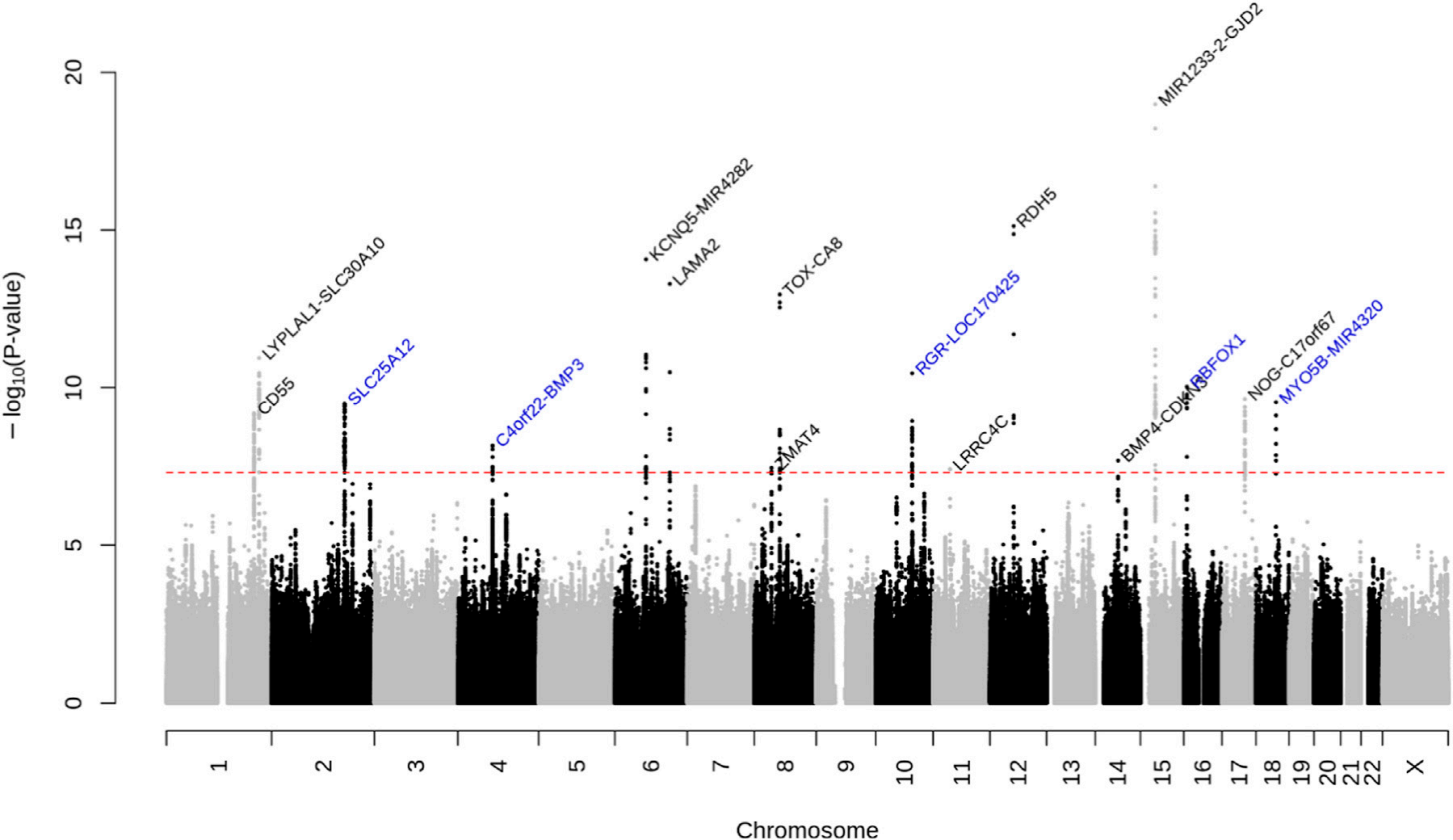
Manhattan plot of the multiethnic ancestry GWA meta-analysis of AL in GERA. The *y*-axis represents the -log_10_(*p*-value); all *p*-values derived from linear regression model are two-sided. The red dotted line represents the threshold of *p* = 5.0 × 10^−8^ which is the commonly accepted threshold of adjustments for multiple comparisons in GWAS. Locus names in blue are for the novel loci and the ones in dark are for the previously reported ones.

We identified 16 loci that reached genome-wide significance in the GERA meta-analysis, of which 5 were novel: *SLC25A12*, near *BMP3*, *RGR*, *RBFOX1*, and *MYO5B* (Figure 1 **;** Supplementary Table S2 **;** Table 2). The genomic inflation factor, λ, of 1.105, is reasonable for a sample of this size (Yang et al., 2011) (Supplementary Figure S1). Regional plots of the association signals at the 5 novel loci are presented in Supplementary Figure S2. These 16 associations with AL were also examined in each individual ethnic group (Supplementary Table S2) and no other SNPs at the 16 loci identified in the multiethnic GWA meta-analysis reached genome-wide level of significance in East Asians, Hispanic/Latinos, or African Americans from the GERA cohort. While the GWAS results generated using REGENIE (Mbatchou et al., 2021) were similar compared to the results generated using PLINK, we found that *MYO5B* was no longer genome-wide significantly associated with AL using REGENIE (lead SNP rs55754534 *p*-value = 3.0 × 10^−7^) (Supplementary Figures S3, S4 **;** Supplementary Table S3). Conducting a GWAS of AL in GERA non-Hispanic white people resulted in the identification of one additional genome-wide significant locus, *PRSS56*, which has been recently reported to be associated with AL in Japanese populations (Fuse et al., 2022) (Supplementary Figures S5, S6 **;** Supplementary Table S4).

**TABLE 2:** Novel AL loci identified in the GERA multiethnic GWA meta-analysis and replication in the CREAM subset.

					Discovery GERA multiethnic meta-analysis				Replication in the (European + Asian ancestry) CREAM subset	
SNP	Chr	Pos	Locus (within or nearest gene)	EA/OA	β (SE)	*P*	*Q*	*I* ^*2*^	β (SE)	*P*
rs57718990	2	172662614	*SLC25A12*	G/T	−0.09 (0.014)	3.26 × 10^−10^	0.79	0	NA	NA
rs1353386	4	81947080	*near BMP3*	C/A	−0.10 (0.016)	6.93 × 10^−9^	0.41	0	−0.035 (0.015)	0.015
rs140405296	10	86019863	*RGR*	AT/ATT	0.09 (0.013)	3.56 × 10^−11^	0.32	14.7	NA	NA
rs58514548	16	7459790	*RBFOX1*	CT/C	0.09 (0.013)	9.46 × 10^−11^	0.28	21.1	NA	NA
rs55754534	18	47433745	*MYO5B*	C/G	0.12 (0.018)	2.96 × 10^−10^	0.45	0	0.04 (0.019)	0.030

Abbreviations: EA, effect allele; OA, other allele; SE, standard error; *I* ^*2*^ *,* heterogeneity index (0%–100%); and *Q, p*-value for Cochrane’s Q statistic.

### Replication in CREAM dataset

Of the 5 novel lead SNPs, two were available in the CREAM dataset. While lead SNP rs1353386 at *C4orf22-BMP3* replicated at a Bonferroni level of significance (*p* ≤ 0.025, 0.05/2 available), SNP rs55754534 at *MYO5B* was nominally associated with AL (*p* = 0.030) in the combined (European and Asian ancestry) CREAM subset (Table 2). In addition, we generated the regional plots at the 5 novel loci using the CREAM European ancestry subset (Supplementary Figure S7), and reported association results for the lead SNPs in CREAM (even if those are different than lead SNPs in GERA) in Supplementary Table S5. Those CREAM results provide evidence of replication at the novel AL identified loci.

### Conditional analysis identified additional loci

Conditional (COJO) analysis in the GERA non-Hispanic white ethnic group revealed eight additional independent signals within the identified genomic regions, including at the *CD55*, *LYPLAL1-SLC30A10*, *HAT1*, *PRSS56*, *C4orf22-BMP3*, *RGR-LOC170425*, *RDH5*, and *MYO5B-MIR4320* loci (Supplementary Table S6).

### Array heritability for AL

We then estimated the array heritability for AL in the GERA non-Hispanic white ethnic group (the largest group of individuals from GERA) using LD score regression (Zheng et al., 2017), and found an overall heritability estimate of 0.37 (s.e. = 0.04).

### Variants prioritization

To prioritize variants within the 15 AL-associated genomic regions identified in the GERA non-Hispanic white ethnic group GWAS analysis, we computed each variant’s ability to explain the observed signal and derived the smallest set of variants that included the causal variant with 95% probability (Chen et al., 2015). The 15 credible sets, corresponding to each of the 15 AL-associated loci, contained from one to 74 variants (355 total variants**,** Supplementary Table S7). Interestingly, two sets included a unique variant (i.e., *KCNQ5-MIR4282* rs7744813, and *LAMA2* rs12193446 with 99.6%, and 99.7% posterior probability of being the causal variants, respectively), suggesting that those variants may be the true causal variants.

### Genes and pathways prioritization

To prioritize genes within the 15 AL-associated genomic regions identified in the GERA non-Hispanic white ethnic group GWAS analysis, we performed a gene-based association analysis using the Versatile Gene-based Association Study (VEGAS2v02) integrative tool (Mishra and Macgregor, 2015). We identified 11 significant genes for AL after correcting for multiple testing (Bonferroni correction was set as *p* < 2.28 × 10^−6^ (0.05/21,950 genes tested)), including *BMP4*, *PRSS56*, and *RGR* (Supplementary Table S8).

We then conducted a pathway analysis using VEGAS2v02 and based on the gene-based association results to assess enrichment in 9,734 pathways (or gene-sets) derived from the Biosystem’s database. We found that pathways involving the cranial skeleton system development as well as the elastic fiber formation were significantly enriched after correcting for multiple testing (Bonferroni correction was set as *p* < 5.14 × 10^−6^ (0.05/9,734 pathways tested)) (Supplementary Table S9).

### Association of lead AL-SNPs with myopic refractive error

**FIGURE 2:**
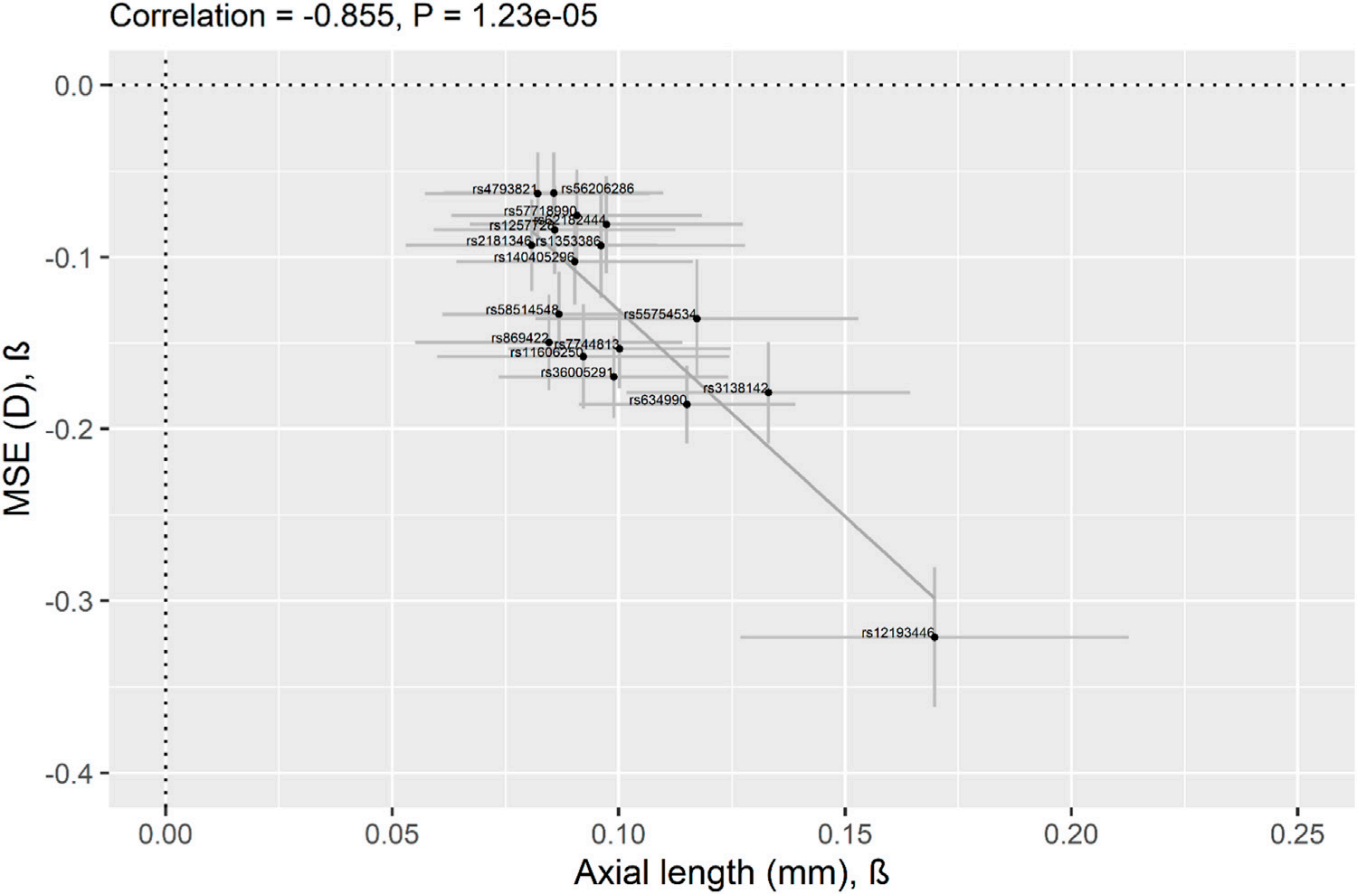
Correlation of effect sizes across studies (AL vs. myopic refractive error) for the lead 17 AL-associated lead SNPs identified in the current study. Comparison of regression coefficients for AL in 19,420 GERA participants (*x*-axis) and for MSE in 72,388 GERA participants (*y*-axis). Pearson’s correlation coefficient = −0.86. Regression Coefficients (betas) are shown, and 95% confidence intervals (CI) are displayed (the horizontal line for each SNP represents the 95% CI of the AL analysis and the vertical line for each SNP represents the 95% CI of the MSE analysis). The 17 AL-associated lead SNPs are shown as black dots and the solid line indicates the line of best fit through the 17 AL lead SNPs.

As previous studies have reported that AL is significantly higher in eyes with myopia compared to normal eyes (Adams, 1987; Grosvenor, 1988; Tideman et al., 2018), we evaluated the effect estimates of the 17 AL-SNPs identified in the current study between AL and MSE in GERA. Our GERA MSE sample consisted of 72,388 participants (Supplementary Table S1). All of the 17 AL-associated SNPs were significantly associated with MSE after Bonferroni correction (*p* < 0.05/17 = 2.94 × 10^−3^), including 15 at genome-wide level of significance (Supplementary Table S10). The Pearson correlation coefficient between AL effect size (beta) and the MSE effect size (beta) was −0.86 (*p* = 1.23 × 10^−5^; Figure 2).

### AL shares genetic determinants with RE and myopia

We also performed genome-wide genetic correlation analyses to quantify genetic overlap between AL and MSE or myopia risk in GERA non-Hispanic white people. We found that AL was genetically correlated with MSE (r_g_ = −0.83; SE, 0.04; *p* = 1.95 × 10^−89^) and myopia (r_g_ = 0.80; SE, 0.05; *p* = 2.84 × 10^−55^). These results suggest considerable shared genetic influences between AL and myopic refractive error.

## Discussion

In this study, by conducting a multiethnic GWA meta-analysis across four ethnic groups, we identified 16 genetic loci for AL, of which 5 were not previously identified. In addition, we reported a genome-wide association at *PRSS56* with AL in non-Hispanic white people. We found that all AL-associated loci were significantly associated with myopic refractive error and observed significant shared genetic effects between AL and MSE or myopia. Finally, we prioritized causal variants, genes, and pathways for AL using bioinformatic functional analyses.

Among the novel GWAS-identified associations with AL, our study revealed potential candidate genes, including *SLC25A12, BMP3*, *RGR*, *RBFOX1,* and *MYO5B*, which have all been linked to visual function or eye development and have been implicated in vision disorders (Kiefer et al., 2013; Stambolian et al., 2013; Verhoeven et al., 2013; Hysi et al., 2020). *SLC25A12* encodes the solute carrier family 25 member 12, which is a calcium-binding mitochondrial carrier protein involved in the exchange of aspartate for glutamate across the inner mitochondrial membrane. Mice lacking *Slc25a12* present with an altered vision (Contreras et al., 2016), as *Slc25a12* plays a pivotal role in retina metabolism providing *de novo* synthesis pathway for glutamine, protects glutamate from oxidation, and is required for efficient glucose oxidative metabolism. Interestingly, our lead AL-associated SNP (rs57718990) at *SLC25A12* has been previously reported to be associated with myopia age-of-onset (Wojciechowski and Cheng, 2018). *BMP3* encodes the bone morphogenetic protein 3, which is a secreted ligand of the TGF-β (transforming growth factor-beta) superfamily of proteins. Polymorphisms at *BMP3* have been previously reported to be associated with RE and myopia (Kiefer et al., 2013; Verhoeven et al., 2013; Hysi et al., 2020), retinal detachment (Boutin et al., 2020), and ocular coloboma (Fox et al., 2022), a congenital disorder characterized by gaps in ocular tissues. BMP3 is found to be localized in the corneal keratocytes and has been proposed to be important for producing and maintaining the extracellular matrix of the corneal stroma (Scott et al., 2011). Our study also identified *RGR*, which encodes a retinal G protein coupled receptor that is largely expressed in intracellular membranes of retinal pigment epithelium and Müller cells (Lin et al., 2007; Zhang and Fong, 2018). A recent study has suggested that light-driven visual cycle that depends on RGR contributes to sustained cone vision under daylight conditions (Morshedian et al., 2019). Mutations in this *RGR* gene have been shown to be associated with retinitis pigmentosa (Morimura et al., 1999; Wang et al., 2001) and different forms of retinal diseases (Li et al., 2016). Our study also implicated *RBFOX1* encoding the RNA binding fox-1 homolog 1, which is expressed in retinal ganglion cells and in amacrine cells of mammalian retinas and has a crucial role in visual function and survival of injured retinal ganglion cells in mice (Gu et al., 2022). Polymorphisms at *RBFOX1* have been shown to be associated with pseudoexfoliation syndrome (Zagajewska et al., 2018). Importantly, polymorphisms at both *RGR* and *RBFOX1* have been previously reported to be associated with refractive error and myopia (Kiefer et al., 2013; Stambolian et al., 2013; Verhoeven et al., 2013; Hysi et al., 2020). Our study also identified *MYO5B* that encodes the myosin VB, involved in the MYO5B-Rab10 system which is required for axon development of vertebrate neocortical neurons or zebrafish retinal ganglion cells *in vivo* (Liu et al., 2013).

Our study also reports, for the first time to our knowledge, a genome-wide association at *PRSS56* with AL in European ancestry individuals. Consistently, a recent GWAS in Japanese populations reported *PRSS56* as an AL-associated locus (Fuse et al., 2022). PRSS56 is a serine protease secreted by retinal Müller glia that has previously been implicated in ocular axial growth (Paylakhi et al., 2018; Koli et al., 2021). *PRSS56* mutations are a major cause of nanophthalmos, characterized by severe shortening of ocular axial length and high hyperopia (Gal et al., 2011; Nair et al., 2011; Orr et al., 2011), and polymorphisms at *PRSS56* have been previously reported to be associated with RE and myopia (Kiefer et al., 2013; Verhoeven et al., 2013; Hysi et al., 2020), angle-closure glaucoma (Nair et al., 2011; Jiang et al., 2013), and ocular abnormalities in humans and mice (Labelle-Dumais et al., 2020; Siggs et al., 2020). Using conditional gene targeting strategies in mice, it has been demonstrated that the loss of PRSS56 function in retinal Müller glia results in a shorter ocular axial length compared to those in control animals (Paylakhi et al., 2018). Furthermore, time-specific inactivation of PRSS56 during post-eye opening period in mice have suggested that sustained PRSS56 activity is required for ocular axial growth during stages including when ocular growth is sensitive to visual input (Paylakhi et al., 2018). Interestingly, retinal *Prss56* expression levels are upregulated in response to lens-induced hyperopic defocus that caused ocular axial elongation/myopia in marmosets suggesting a potential of Prss56 in emmetropization, which is avision-guided process ocular growth (Tkatchenko et al., 2018).

Our multiethnic GWAS of AL identified 2 previously reported loci (*ZMAT4* and *BMP4-CDKN3*) that did not reach genome-wide level of significance in our non-Hispanic white GWAS. Interestingly, both *ZMAT4* and *BMP4-CDKN3* have been recently identified as AL-associated loci in a recent GWAS conducted in Japanese individuals (Fuse et al., 2022). Common genetic variants at those *ZMAT4* and *BMP4-CDKN3* loci have been previously reported to be associated with MSE, and high myopia, especially in Asian populations (Yoshikawa et al., 2014; Cheong et al., 2020). More recently, a whole exome sequencing study for refractive error conducted in 51,624 unrelated adults of European ancestry identified novel putative causal variants in the *BMP4* gene (Guggenheim et al., 2022).

In our study, all AL-associated loci were significantly associated with myopic refractive error and we also observed significant genetic correlations between AL and MSE or myopia. Interestingly, a recent GWAS of corneal curvature, which serves as a proxy for eye size, identified 32 loci and revealed a lack of genetic correlation between eye size and refractive error (Plotnikov et al., 2021). This suggests that genetic variants associated with human eye size are distinct from those conferring susceptibility to myopia. In this recent GWAS of corneal curvature (Plotnikov et al., 2021), the authors also reported significant genetic correlations between corneal curvature and eye size and between eye size and body height. Thus, AL and corneal curvature are two distinct traits and while AL is a relevant endophenotype for refractive error and myopia, corneal curvature reflects more eye size and body height.

Our bioinformatic annotation analyses revealed potential biological pathways involved in AL variation that are relevant to the embryonic cranial skeleton morphogenesis and the elastic fibers formation, consistent with previous works (Ouyang et al., 2019). Overall, the identified genes suggest a role for extracellular matrix remodeling, visual cycle and neuronal development in the determination of AL, similar to those previously suggested to participate in myopia pathogenesis (Kiefer et al., 2013). Follow-up functional experiments utilizing animal models could confirm the involvement of these biological pathways in AL variation and provide insights into the underlying mechanisms of AL-related vision disorders such as myopia.

We recognize potential limitations of our study. First, our study was limited by its restriction to common variants (minor allele frequency ≥1%), which did not allow us to identify lower frequency variants that contribute to variation in AL. Future whole-exome sequencing studies could identify less common variants associated with AL. Second, in our study, we note that the Hispanic/Latino, East Asian, and African American subgroups had smaller sample sizes compared to the non-Hispanic white ethnic group, potentially limiting statistical power to detect some SNP associations in those groups. Future large and ethnically diverse studies will be needed to uncover the genetic architecture of AL in those ethnic groups.

In summary, our large multiethnic GWA meta-analysis provides new insights into the genetic architecture of AL. In addition to expanding the list of AL-associated loci, this study also provides evidence of shared genetic etiology between AL and MSE, or myopia. Altogether, study findings may open new avenues of investigation into eye biometry and its relationship to the risk of vision disorders, such as myopia.

## Data Availability

The multiethnic GERA GWAS summary statistics are available from the NHGRI-EBI GWAS Catalog (https://www.ebi.ac.uk/gwas/downloads/summary-statistics). The GERA genotype data are available upon application to the KP Research Bank (https://researchbank.kaiserpermanente.org/). Pathways or gene-sets were derived from the Biosystem’s database which can be accessed through the following link (https://vegas2.qimrberghofer.edu.au/biosystems20160324.vegas2pathSYM).
