# Supplementary material for: A multiethnic genome-wide analysis of 19,420 individuals identifies novel loci associated with axial length and shared genetic influences with refractive error and myopia
Source: Front Genet. 2023 Jun 7;14:1113058. doi: 10.3389/fgene.2023.1113058 (PMC10282939; doi:10.3389/fgene.2023.1113058)
Supplement: Supplementary file 1 [file Presentation1.PDF]

Jiang et al. "A multiethnic genome-wide analysis of 19,420 individuals identifies novel loci associated with axial length and shared genetic influences with refractive error and myopia"

Supplementary Figures

Supplementary Figure S1. QQ plot and genomic inflation factor (λ) observed for the multiethnic GWA meta-analysis of AL in GERA.

Supplementary Figure S2. Locus Zoom plots of novel regions identified in the GERA multiethnic GWA meta-analysis of AL.

Supplementary Figure S3. QQ plot and genomic inflation factor (λ) observed for the multiethnic GWA meta-analysis of AL in GERA using REGENIE.

Supplementary Figure S4. Manhattan plot of the GERA multiethnic GWA meta-analysis of AL using REGENIE.

Supplementary Figure S5. QQ plot and genomic inflation factor (λ) observed for the non-Hispanic white GWAS of AL in GERA.

Supplementary Figure S6. Manhattan plot of the non-Hispanic white GWAS of AL in GERA.

Supplementary Figure S7. Locus Zoom plots of novel regions based on CREAM European ancestry subset GWAS results.

Supplementary Figure S1. QQ plot and genomic inflation factor (λ) observed for the multiethnic GWA meta-analysis of AL in GERA

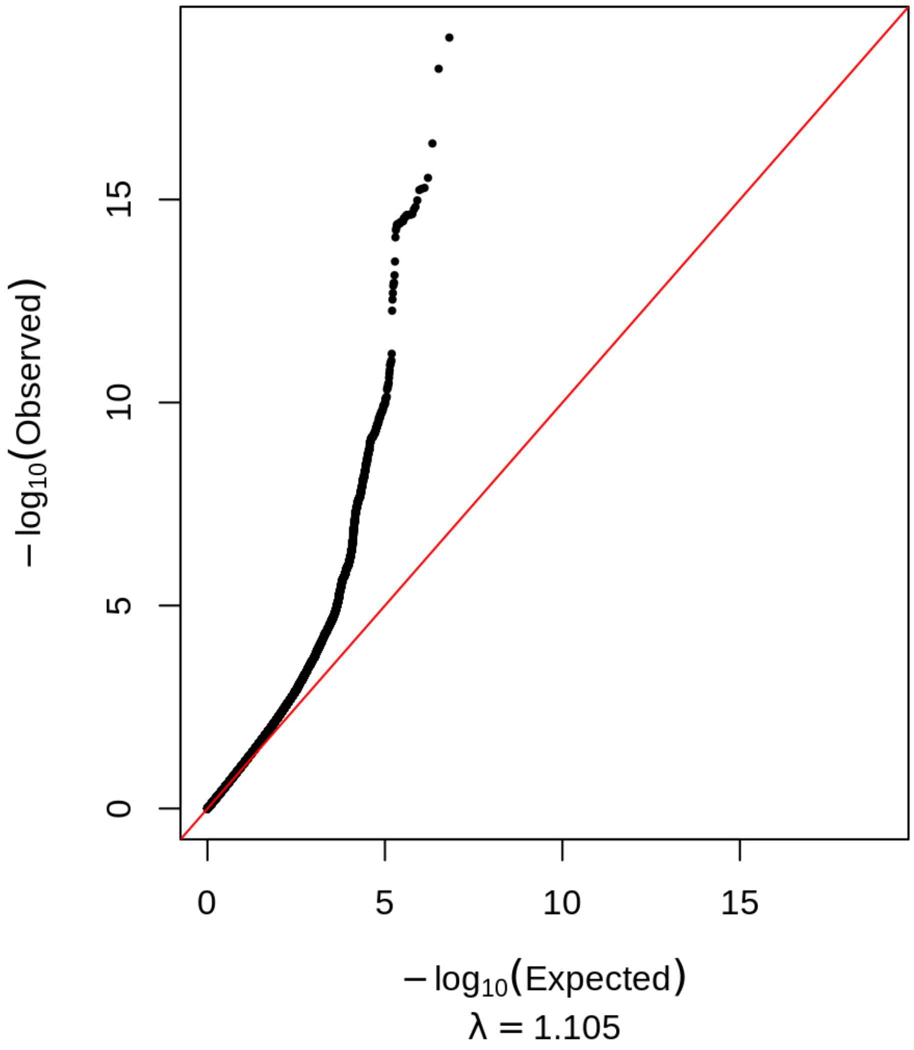

Supplementary Figure S2. Locus Zoom plots of novel regions identified in the GERA multiethnic GWA meta-analysis of AL.

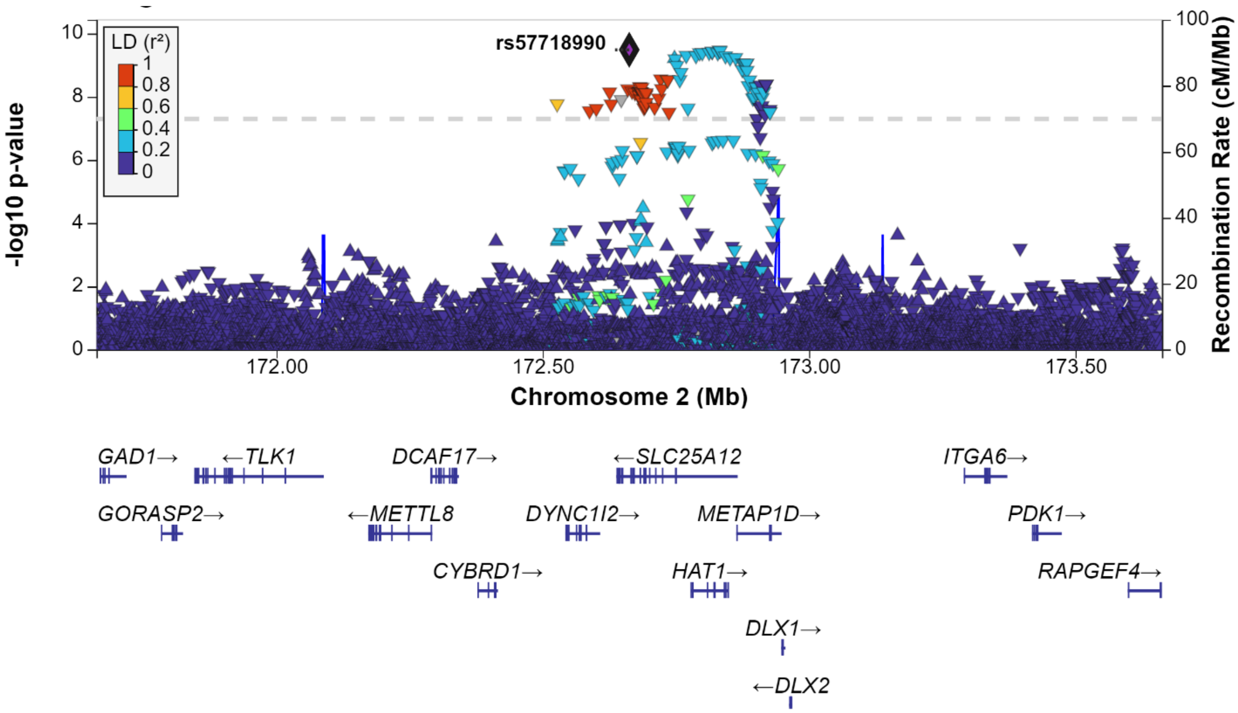

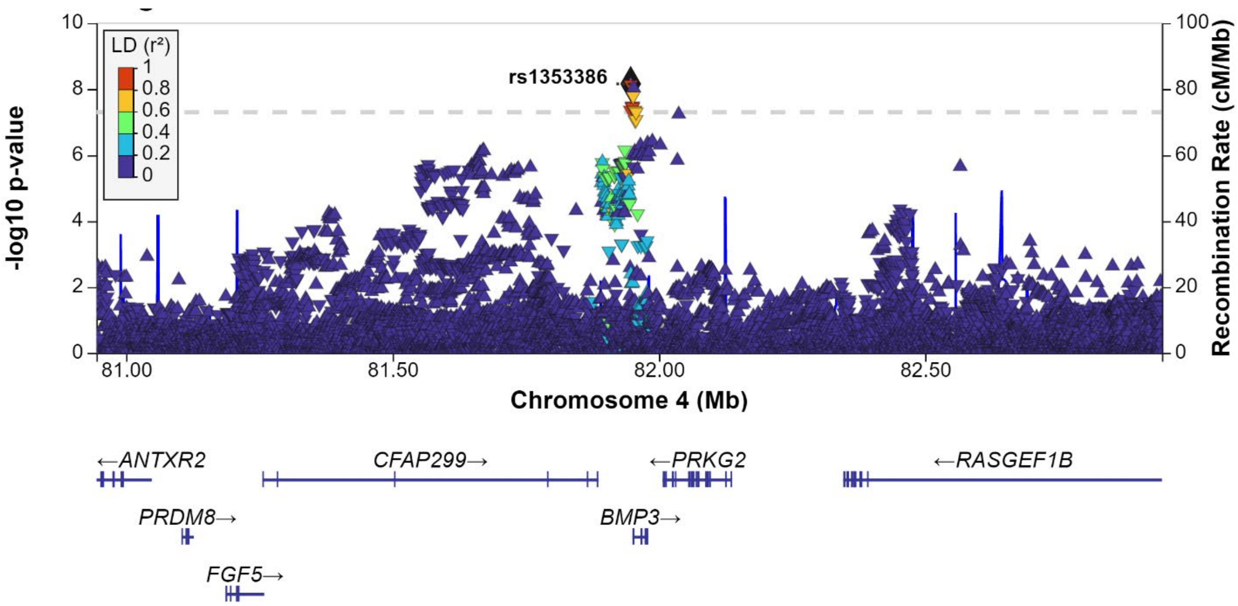

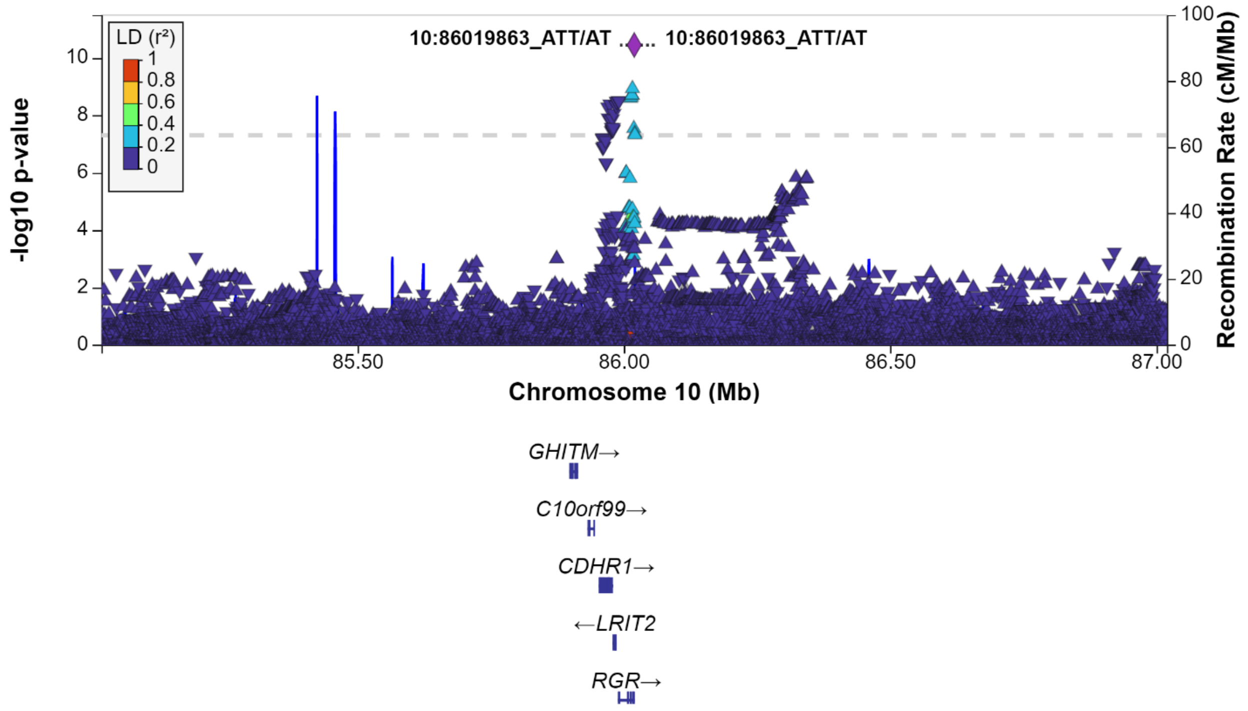
LD (r²)
1
0.8
0.6
0.4
0.2
0
10:86019863_ATT/AT
10:86019863_ATT/AT
-log10 p-value
Recombination Rate (cM/Mb)
Chromosome 10 (Mb)
85.50
86.00
86.50
87.00
GHITM→
C10orf99→
CDHR1→
←LRIT2
RGR→

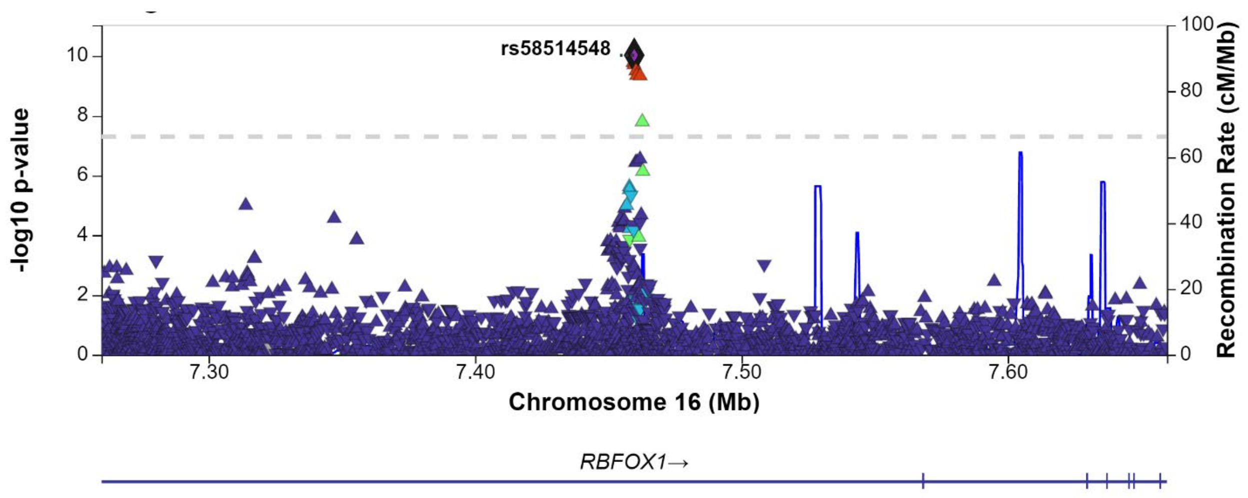
rs58514548
-log10 p-value
Recombination Rate (cM/Mb)
Chromosome 16 (Mb)
7.30
7.40
7.50
7.60
RBFOX1→

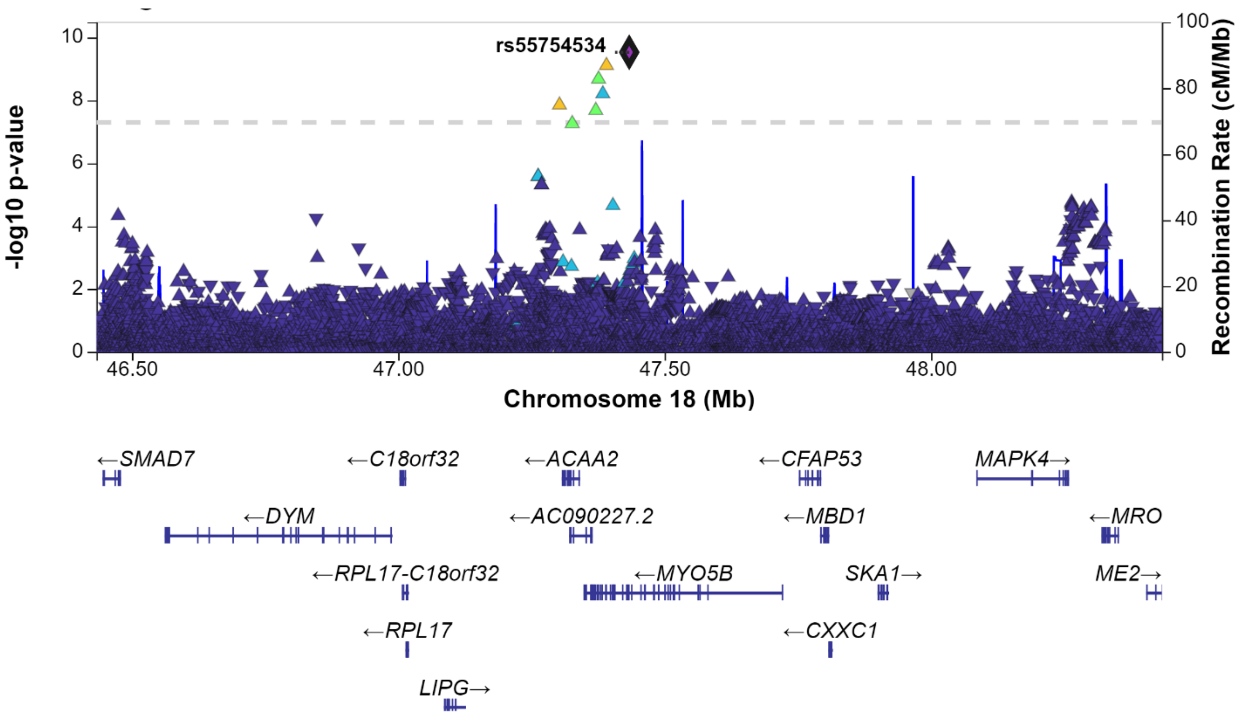
rs55754534
-log10 p-value
Recombination Rate (cM/Mb)
Chromosome 18 (Mb)
46.50
47.00
47.50
48.00
←SMAD7
←C18orf32
←ACAA2
←CFAP53
MAPK4→
←DYM
←AC090227.2
←MBD1
←MRO
←RPL17-C18orf32
←MYO5B
SKA1→
ME2→
←RPL17
←CXXC1
LIPG→

Supplementary Figure S3. QQ plot and genomic inflation factor (λ) observed for the multiethnic GWA meta-analysis of AL in GERA using REGENIE.

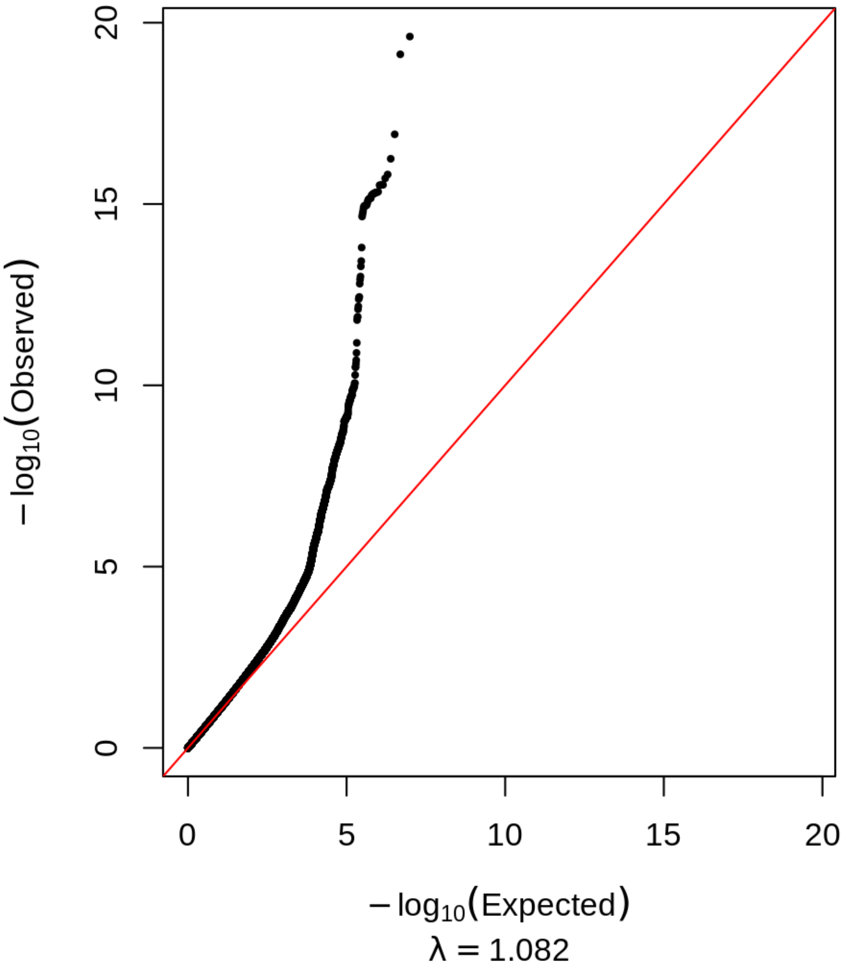

Supplementary Figure S4. Manhattan plot of the GERA multiethnic GWA meta-analysis of AL using REGENIE. The y-axis represents the -log10(P-value); all P-values derived from linear regression model are two-sided. The red dotted line represents the threshold of P=5x10^{-8} which is the commonly accepted threshold of adjustments for multiple comparisons in GWAS. Locus names in blue are for the novel loci and the ones in dark are for the previously reported ones.

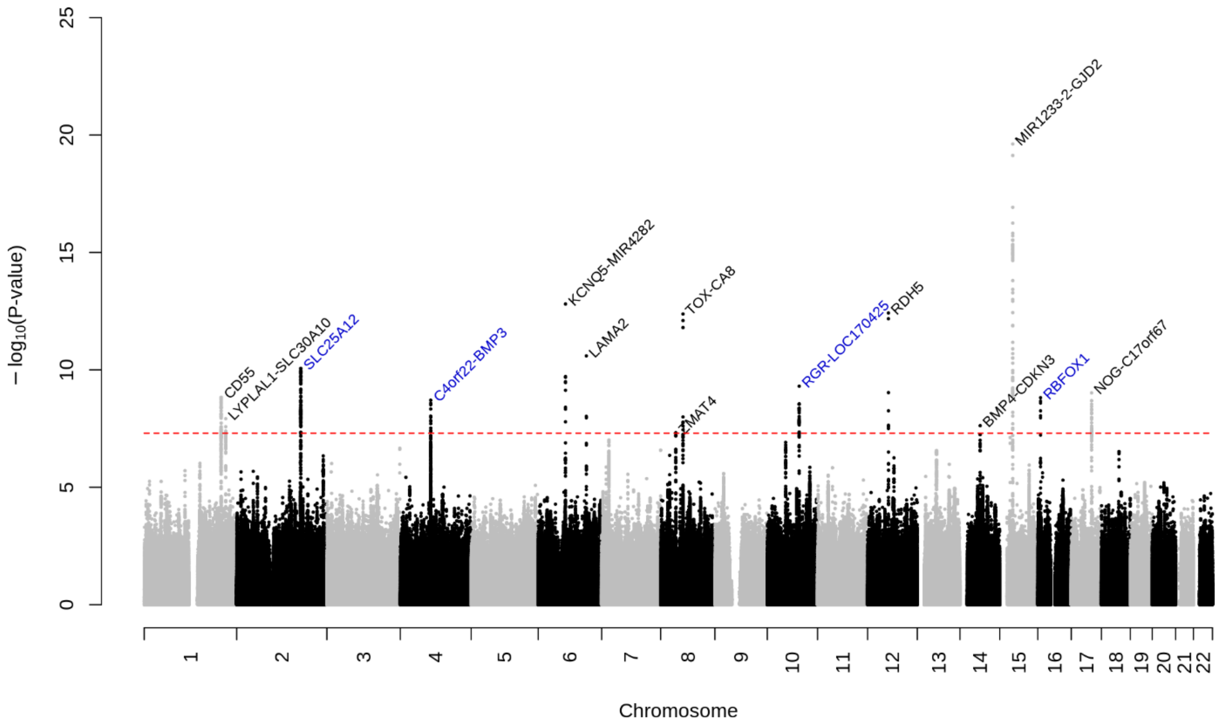

Supplementary Figure S5. QQ plot and genomic inflation factor (λ) observed for the non-Hispanic white GWAS of AL in GERA.

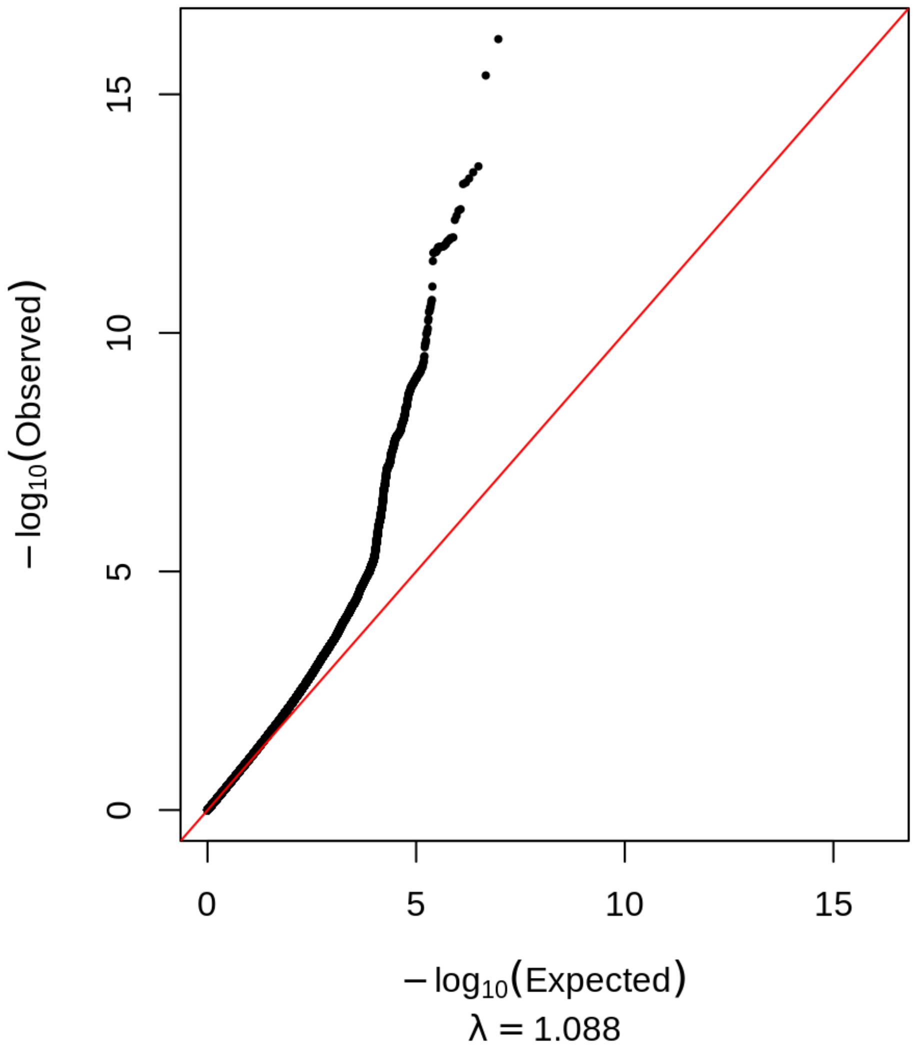

Supplementary Figure S6. Manhattan plot of the non-Hispanic white GWAS of AL in GERA. The y-axis represents the -log10(P-value); all P-values derived from linear regression model are two-sided. The red dotted line represents the threshold of P=5x10^{-8} which is the commonly accepted threshold of adjustments for multiple comparisons in GWAS. Locus names in blue are for the novel loci and the ones in dark are for the previously reported ones.

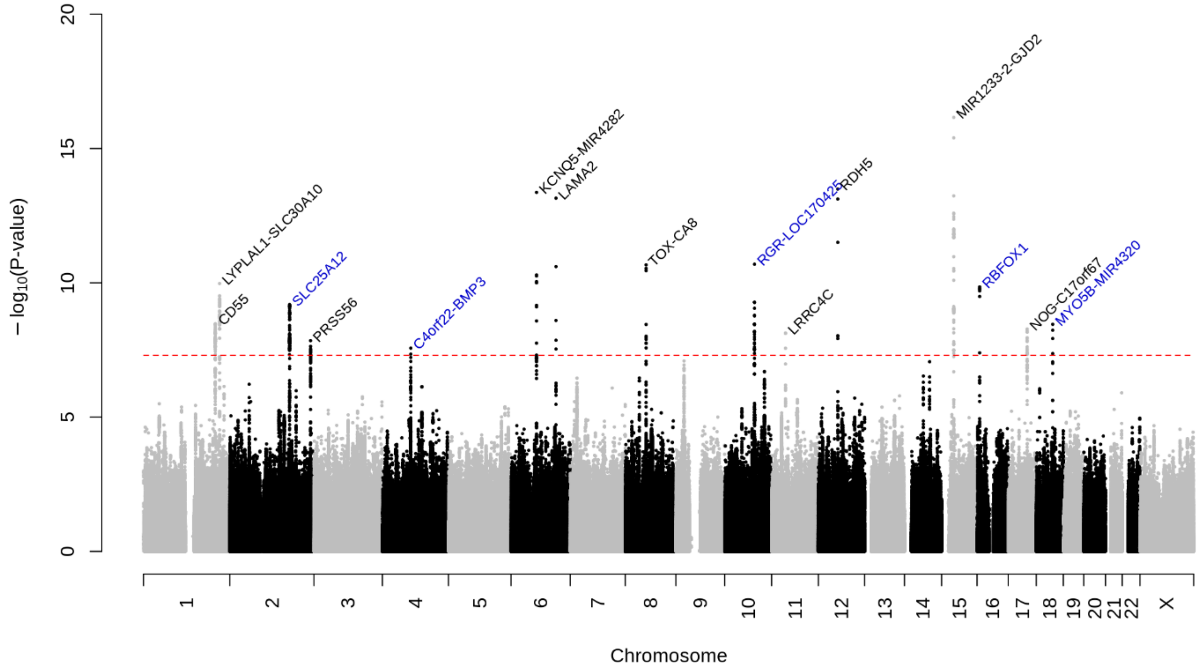

Supplementary Figure S7. Locus Zoom plots of novel regions based on CREAM European ancestry subset GWAS results.

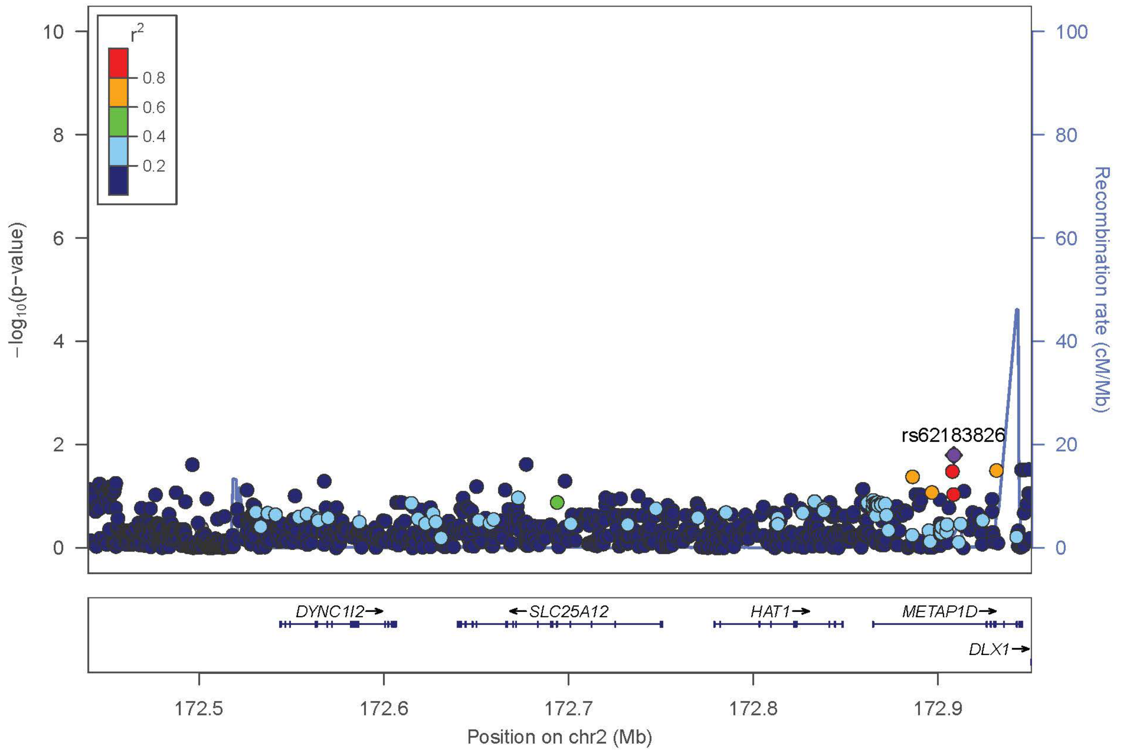

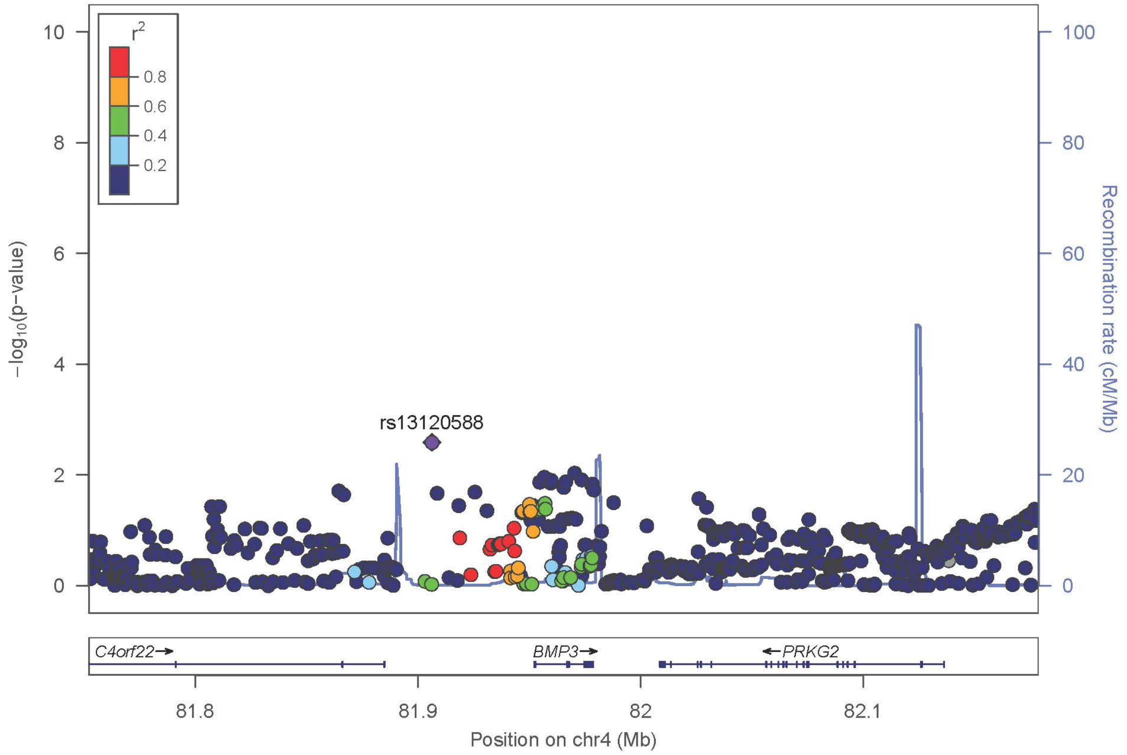

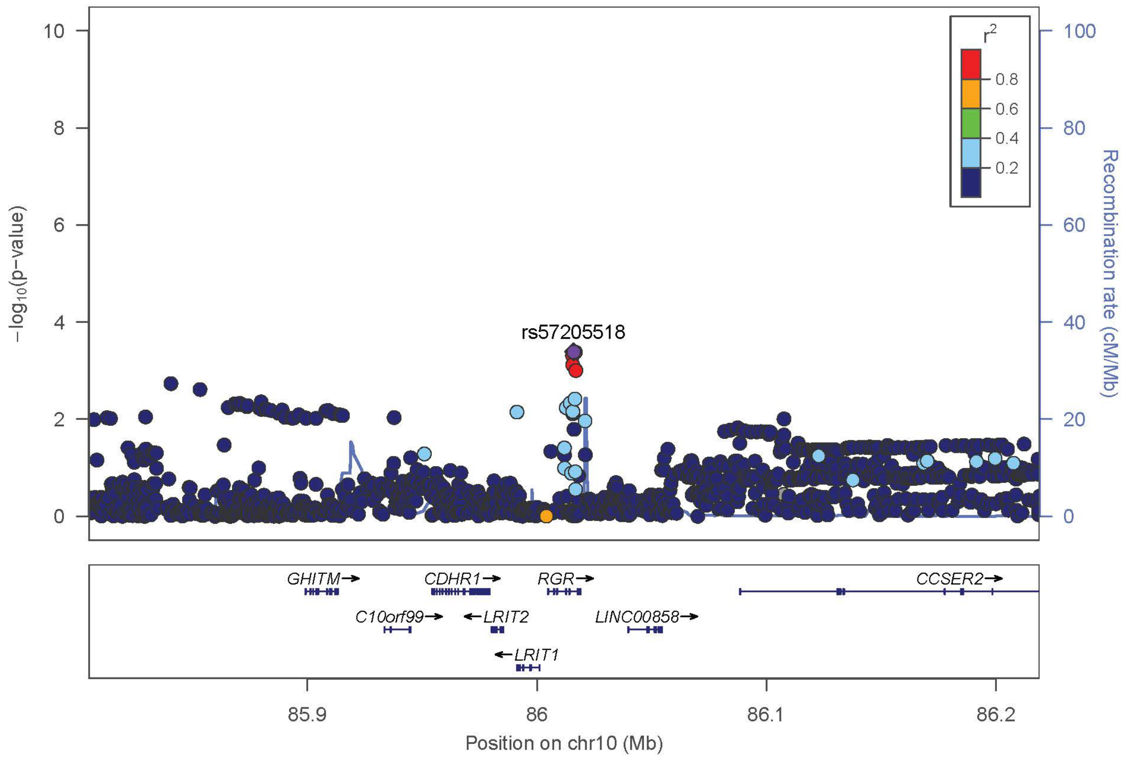

rs57205518
r2
0.8
0.6
0.4
0.2
−log10(p−value)
Recombination rate (cM/Mb)
GHITM
CDHR1
RGR
CCSER2
C10orf99
LRIT2
LINC00858
LRIT1
85.9
86
86.1
86.2
Position on chr10 (Mb)

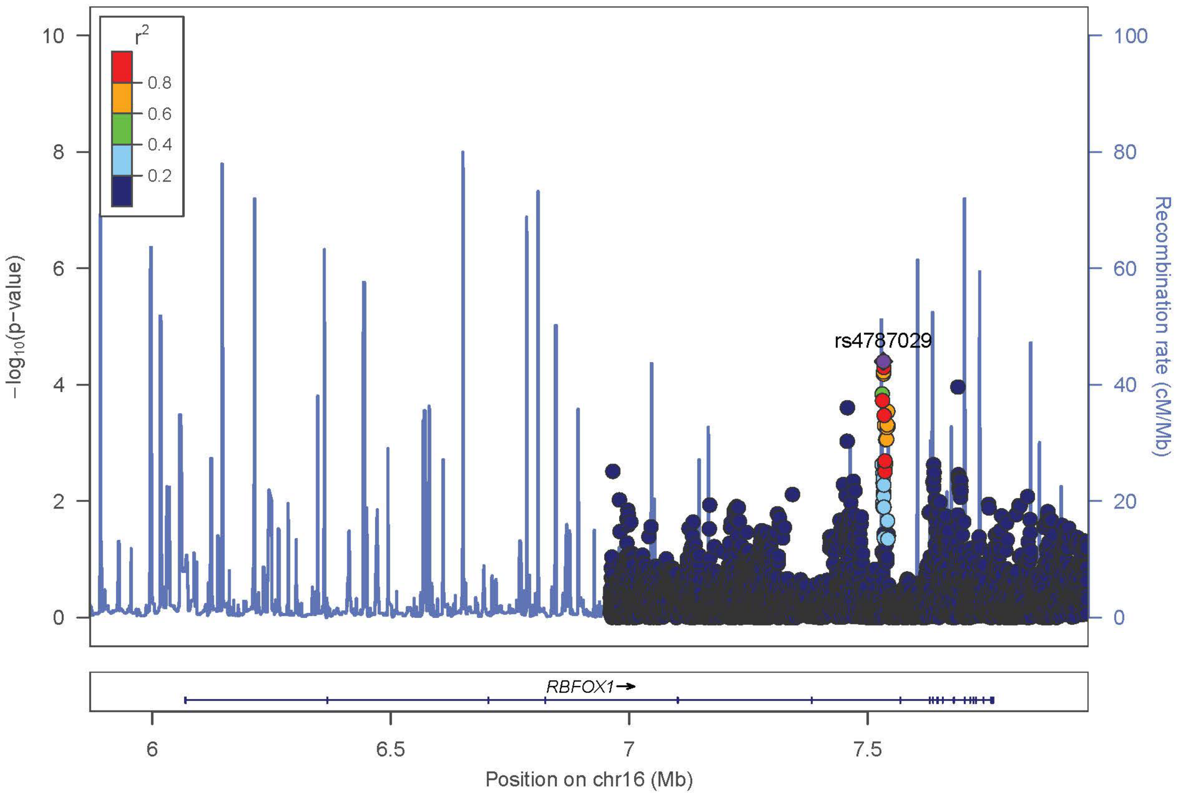

rs4787029
r2
0.8
0.6
0.4
0.2
−log10(p−value)
Recombination rate (cM/Mb)
RBFOX1
6
6.5
7
7.5
Position on chr16 (Mb)

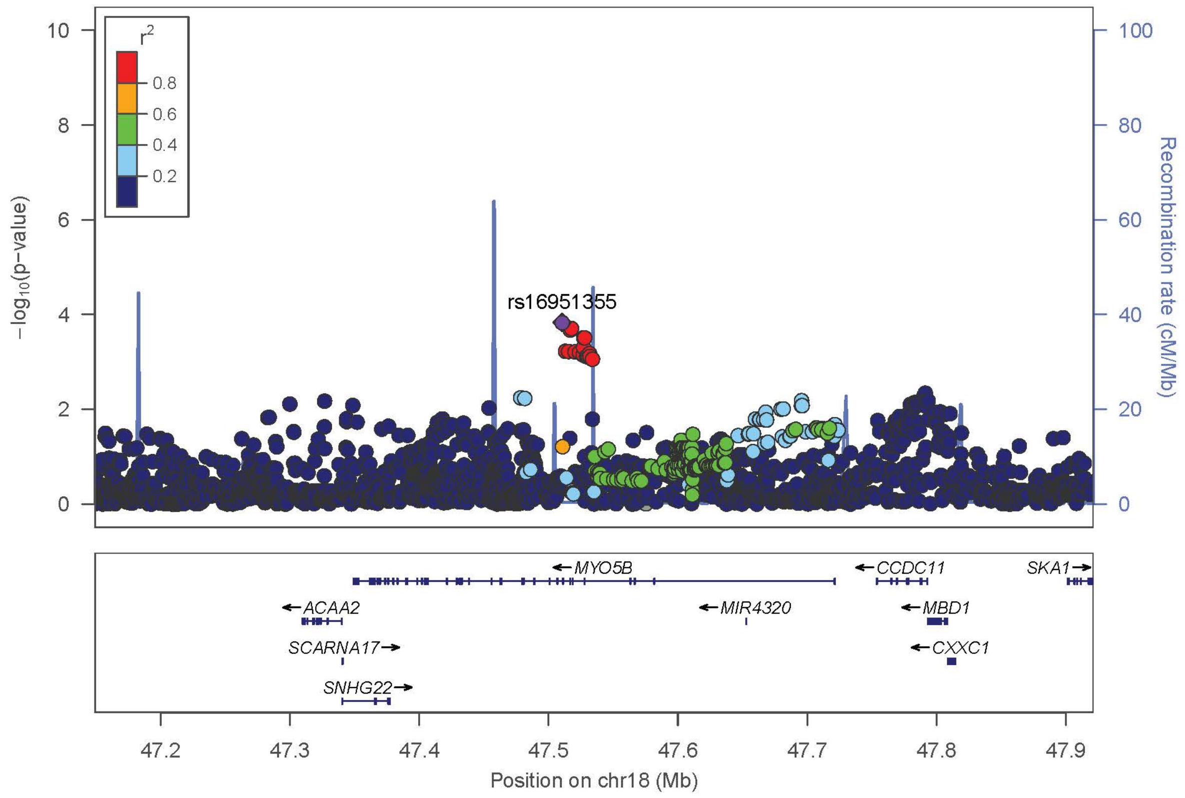

r2
0.8
0.6
0.4
0.2
rs16951355
−log10(p-value)
Recombination rate (cM/Mb)
10
8
6
4
2
0
100
80
60
40
20
0
MYO5B
CCDC11
SKA1
ACAA2
MIR4320
MBD1
SCARNA17
CXXC1
SNHG22
47.2
47.3
47.4
47.5
47.6
47.7
47.8
47.9
Position on chr18 (Mb)
